# Supplementary material for: Phase-selective in-plane heteroepitaxial growth of H-phase CrSe2
Source: Nat Commun. 2024 Feb 26;15:1765. doi: 10.1038/s41467-024-46087-0 (PMC10897461; doi:10.1038/s41467-024-46087-0)
Supplement: Supplementary file 1 — Supplementary Information [file 41467_2024_46087_MOESM1_ESM.pdf]

Supplementary Information: Phase-selective in-plane heteroepitaxial growth of H-phase $CrSe_2$

Meizhuang Liu[1,2]*, Jian Gou[2,3], Zizhao Liu[4], Zuxin Chen[5], Yuliang Ye[5], Jing Xu[5], Xiaozhi Xu[1], Dingyong Zhong[4], Goki Eda[2], Andrew T. S. Wee[2]*

[1]*School of Physics, Guangdong Basic Research Center of Excellence for Structure and Fundamental Interactions of Matter, Guangdong Provincial Key Laboratory of Quantum Engineering and Quantum Materials, South China Normal University, Guangzhou, 510006, China*
[2]*Department of Physics, National University of Singapore, 2 Science Drive 3, 117542, Singapore*
[3]*School of Physics, Zhejiang University, Hangzhou, 310027, China*
[4]*School of Physics and State Key Laboratory of Optoelectronic Materials and Technologies, Sun Yat-sen University, Guangzhou, 510275, China*
[5]*School of Semiconductor Science and Technology, South China Normal University, Guangzhou, 510631, China*

Supplementary Notes

1. MBE growth and STM characterization of $MoSe_2$ on the HOPG substrate

The growth temperature has been demonstrated to be one of the key factors in the MBE growth of $MoSe_2$ nanoribbons by previous works[1,2]. When the substrate temperature is lower than 250 °C, the MBE-grown $MoSe_2$ flakes have fractal shapes (as shown in Fig. S1**a**) due to the lower mobility of adatoms around the island edges. As the temperature is elevated, 2D $MoSe_2$ islands with higher crystallinity can be obtained (Fig. S1**b**). Meanwhile, the density and length of MTBs in the $MoSe_2$ monolayers can be controlled by tuning the growth temperature. More isolated and longer MTBs will be got at higher temperature (Fig. S1**d**). In the long MTBs, the STM instensity modulations can be observed with nonuniform amplitudes along the MTB. As shown in the Fig. S1**d**, the modulations are strongest near the edge of MTBs and decrease gradually from the edge to the middle postion of MTBs. The behavior can be decribed by the Friedel oscillations[4]. The $MoSe_2$ nanoribbons with well-defined orientations form at the substrate temperature kept at about 550 °C (Fig. S1**e**). The monolayer and bilayer $MoSe_2$ nanoribbons with straight edges obtained in our experiments are shown in Fig. S1**e**. Based on the previous study of $MoSe_2$ nanoribbons[1,3], the lefe edge of the $MoSe_2$ nanoribbon in the close-up STM image (Fig. S1**f**) can be identified as the Se-edge and the right edge is the Mo-edge.

Apart from the growth temperature, the Se:Mo flux also has the equivalent effects on the growth favouring nanoribbon growth at lower Se concentrations. The evolution of $MoSe_2$ island shapes is determined by the relative energies and growth rates of the different edge structures. The atomic growth mechanism of $MoSe_2$ nanoribbon has been revealed by DFT calculations in the reported work[1]. The armchair edges were calculated to have the higher energy than the zigzag (Mo- and Se- terminated) edges. Therefore, the armchair edges grow much faster than the zigzag edges, which results in the ribbon structures.

The width control of MBE-grown $MoSe_2$ nanoribbons through growth temperature has been demonstrated by previous works, in which the width will gradually decrease as the growth temperature is elevated[2]. The same tendency in the MBE growth of $MoSe_2$ nanoribbons can also be observed in our experiments. The thickness can be controlled by tuning the growth duration. The density control of $MoSe_2$ nanoribbons can be achieved by tuning the growth parameters of flux rate and growth duration. At the optimized growth parameters of growth temperature and Se:Mo flux, the 1D $MoSe_2$ nanoribbons are able to be controlled at the monolayer level by tuning the flux rate and growth duration. Regarding to $MoSe_2$ nanoribbons grown at 550 °C in our experiments, the statistical analysis of width and layer numbers was carried out (Fig. S1**g** and 1**h**). The widths of $MoSe_2$ nanoribbons are mostly distributed from 10 to 25 nm. The statistical analysis on thickness distribution indicates the $MoSe_2$ nanoribbons are mostly monolayer and bilayer. The band profile of the monolayer $MoSe_2$ nanoribbon with a length of ~20 nm is directly visualized by the 2D plot of dI/dV spectra across the nanoribbon (Fig. S1**j**). The edge states can be observed at the edge termination due to the existence of dangling bongs. The upward band bending near the edge can be ascribed to the dangling electron (hole) states, which is consistent with the previous report[5].

2. STM characterization of subsequently grown chromium selenide at different growth temperatures and deposition duration

In our experiment, Cr and Se atoms are codeposited onto the HOPG substrate after successful preparation of $MoSe_2$ nanoribbons. Excessive selenium atoms were sublimed to maintain the Se-rich environment during the growth. The lowest growth temperature was set at 120 °C for thermally desorbing the excessive Se atoms from the substrate. At the grow temperature of 120 °C, only low-quality film of chromium selenide can be obtained (as shown in Fig. S2**a**). Thermal energy is essential for atoms diffusing and nucleating to form the better films. However, when the growth temperature is higher than 300 °C, most samples are the Cr_2Se_3, whose heights are 0.3 nm higher than that of $MoSe_2$ (Fig. S2**b**). Thereofore, the proper substrate temperature was chosen at 180-250°C to grow 1H-$CrSe_2$. When we deposite a small amount of Cr atoms, the $MoSe_2$-$CrSe_2$ lateral heterostructures accompanied with narrow-width $CrSe_2$ nanoribbons can be obtained (Fig. S2**c** and **d**). When we increase the deposition

duration, the isolated $CrSe_2$ islands grown on the top of $MoSe_2$ can be observed (as shown in Fig. S2**e** and **f**). Most samples are the 1T phase with non-layered structures.

3. Mechanism exploration of the in-planed template induced selective growth of H-phase $CrSe_2$

When the spin polarization and Coulomb interaction were considered, the T-phase $CrSe_2$ is calculated to have a larger lattice constant and a lower energy than the H-phase structure. However, as the lattice is restricted by the in-plane template of $MoSe_2$ nanoribbons (3.3A), the H-phase $CrSe_2$ is calculated to be the more stable configuration. To uncover the effect of in-plane epitaxial template, DFT calculations were carried out to simulate the binding energies of different numbers of H-phase and T-phase $CrSe_x$ radicals combined at the Se-edge of the $MoSe_2$ nanoribbon (Fig. S3**a**). The H-phase radicals possess the larger binding energies compared with the result of T-phase ones. The DFT calculations indicate that the formation of H-phase radicals at the edges of $MoSe_2$ nanoribbons is energetically preferred. As the number of the $CrSe_x$ radical increases and the radicals nucleate at the edges, the heterostructures with the 1H-1H or 1H-1T interface structures can be formed. Compared with the simulated interfacial structures between 1T-$CrSe_2$ and 1H-$MoSe_2$ monolayers, the H-phase $CrSe_2$ structures connected to the Mo-edge and Se-edge of $MoSe_2$ nanoribbon are demonstrated to be the more stable configuration than the T-phase structures (Fig. S3**b**). The continuity of 1H phase structures can avoid the occurrence of 1H-1T structures with higher interfacial energy.

4. Tomonaga−Luttinger liquid (TLL) behavior in the (quasi) one-dimensional metallic MTBs

For 2D or 3D metallic systems, the electronic behavior can be described by Laudau Fermi liquid (FL) theory of non-interacting quasiparticles. When the electrons are confined in 1D systems, the quasiparticle excitation mechanism breaks down and electrons become a strongly correlated quantum liquid obeying the Tomonaga-Luttinger liquid (TLL) behavior[6,7]. As (quasi) one-dimensional metallic systems, TLL behavior has been revealed in the MTBs. As shown in Fig. S9**b**, the gap size increases with the length of MTBs getting shorter, which is the signature of TLL behavior. In the TLL theory, the energy gap of the finite system with length L can be described as $E_{gap} = [(\pi v_c/2K_c) + (\pi v_s/2K_s)](1/L)$, where v_c and v_s stand for the velocity of charge and spin excitation, respectively. Two Luttinger parameters K_c and K_s encode the interaction strength. Another signature of TLL behavior is the spin-charge separation which has the distinct dispersions of spin and charge excitations with velocities v_s and v_c. In the Fourier transformation of 2D plot of dI/dV spectra which can directly reveal the dispersion of confined states, two linear dispersion branches with different slopes corresponding to the spin and charge density excitations can be observed.

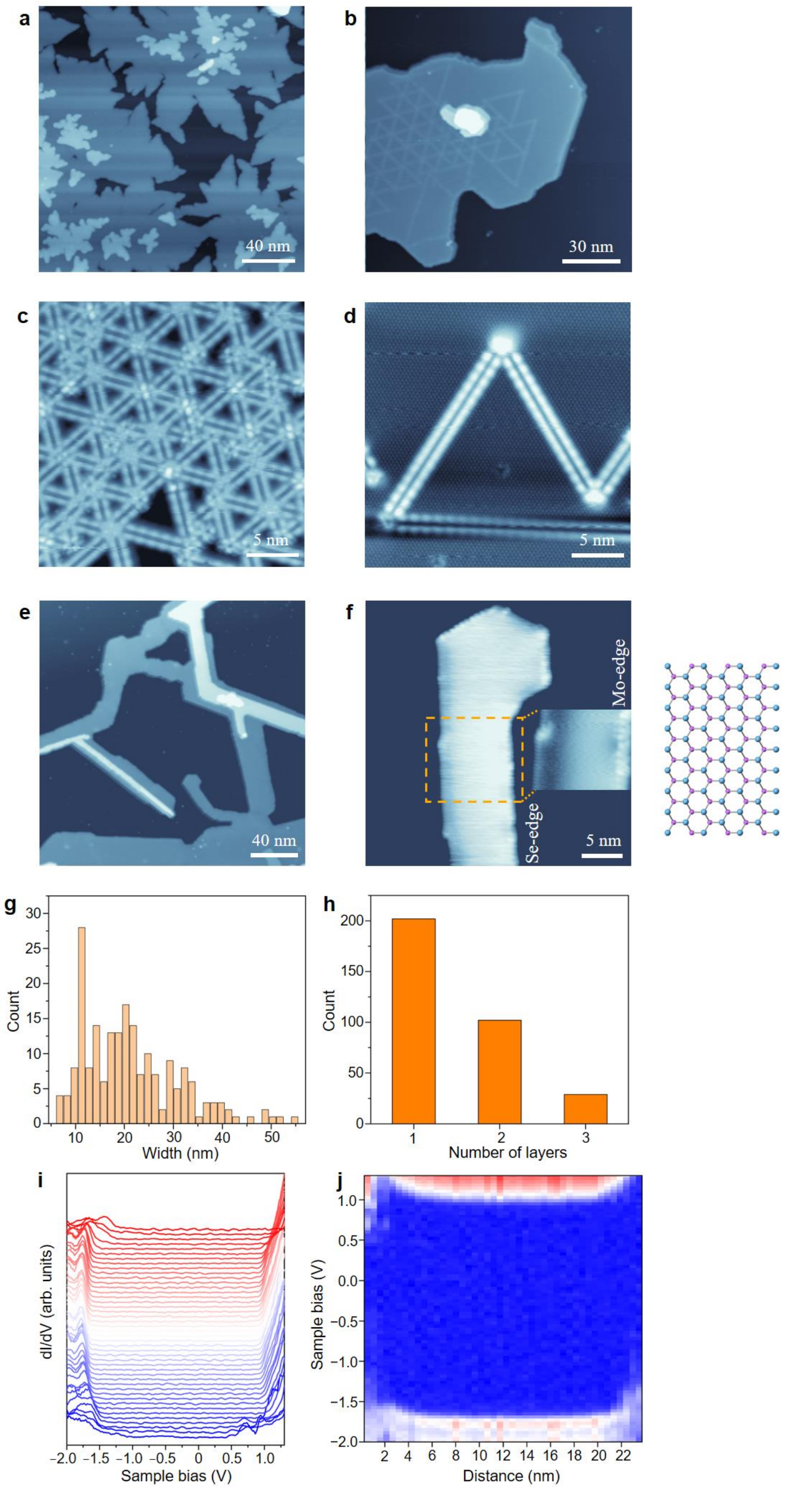

Supplementary Figure 1 | MBE growth and STM characterization of $MoSe_2$ on the HOPG substrate. a,**b**, STM image of the $MoSe_2$ monolayer grown at about 250 °C and 400 °C. **c**,**d**, The corresponding atomic-resolution STM image of MTBs in **a** and **b** ($V_S = -0.8$ V, $I_t = 300$ pA, 500 pA). **e**, STM image of $MoSe_2$ nanoribbons with straight edges ($V_S = 2$ V, $I_t = 10$ pA). **f**, Close-up STM image of the $MoSe_2$ nanoribbon with the corresponding atomic model shown in right panel. Statistical analysis of ribbon width (**g**) and thickness (**h**) of $MoSe_2$ grown at 550 °C. **i**, Position-dependent dI/dV spectra acquired across the nanoribbon. **j**, 2D plot of the dI/dV spectra across the nanoribbon.

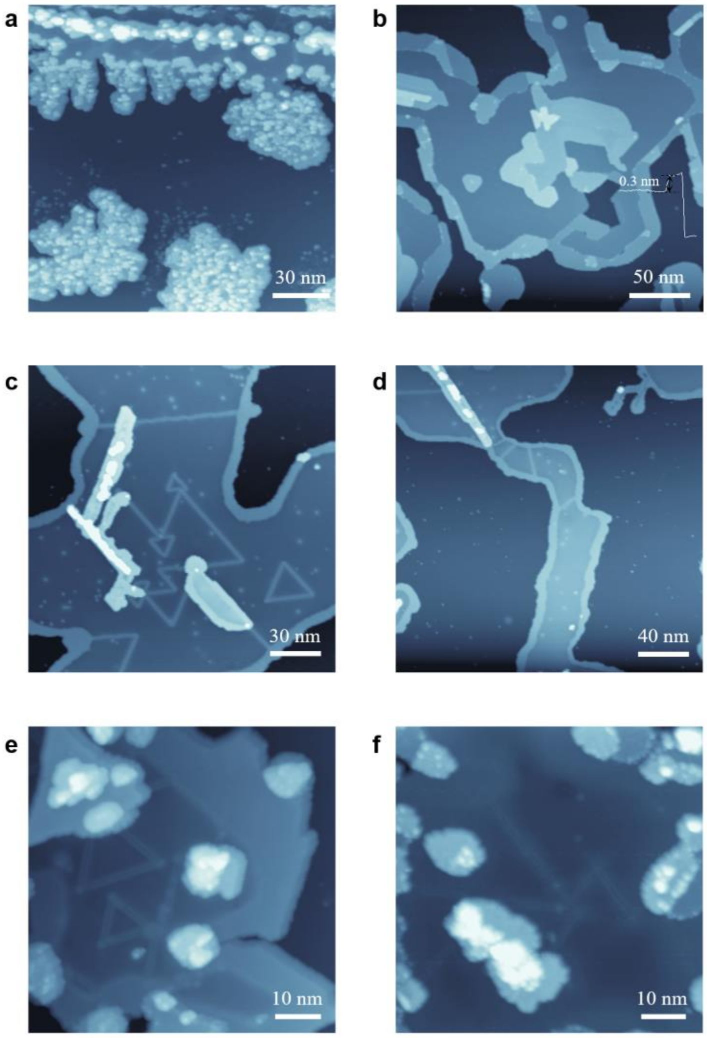

Supplementary Figure 2 | STM characterization of subsequently grown chromium selenide at different growth temperatures and deposition duration. a, STM image of chromium selenide grown at 120 °C (V_S = −2 V, I_t = 10 pA). **b**, STM image of $MoSe_2$ together with Cr_2Se_3 islands grown at 350 °C (V_S = −2.2 V, I_t = 8 pA). **c**,**d**, STM images of $MoSe_2$-$CrSe_2$ lateral heterostructure with a small amount of $CrSe_2$ grown at 200 °C (V_S = 1.3 V, I_t = 10 pA). **e**, **f**, STM images of isolated 1T-$CrSe_2$ islands grown on the top of $MoSe_2$ (V_S = −1.8 V, I_t = 30 pA).

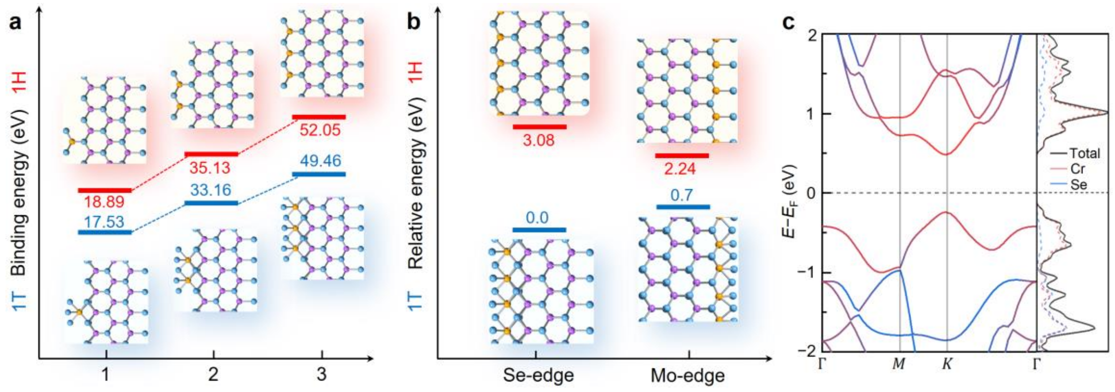

Supplementary Figure 3 | Growth mechanism exploration of the in-planed template induced phase-selective synthesis of H-phase $CrSe_2$. a, DFT calculated binding energies of different numbers of H-phase and T-phase $CrSe_x$ radicals combined at the Se-edge of the $MoSe_2$ nanoribbon. **b**, Theoretical calculation of relative energies of the 1H-1H and 1H-1T interface structures at the Se-edge and Mo-edge of $MoSe_2$ nanoribbon, respectively. **c**, DFT calculated band structure and corresponding density of state of monolayer H-phase $CrSe_2$. The colour reflects the character of the states with red corresponding to chromium and blue to selenium.

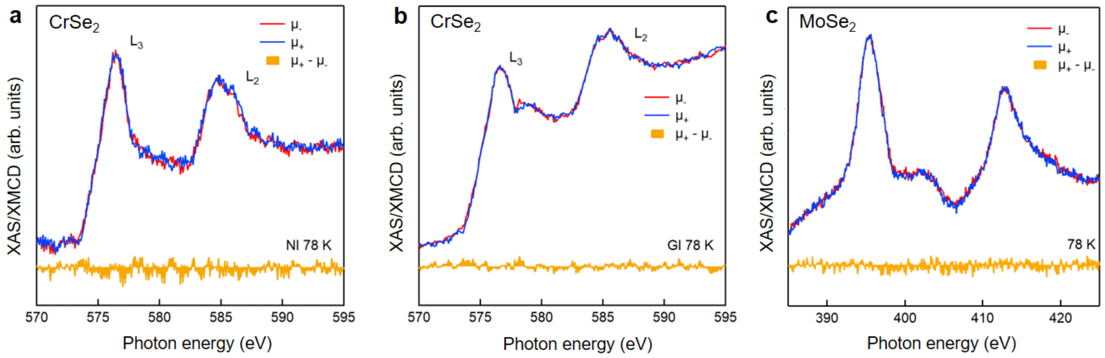

Supplementary Figure 4 | XAS/XMCD spectra of the H-phase $CrSe_2$ and $MoSe_2$ at 78 K. XAS/XMCD spectra of H-phase $CrSe_2$ measured in the normal incidence (NI) **a** and grazing incidence (GI) **b** directions at 78 K. The XMCD signal, $\Delta\mu = \mu_{+} - \mu_{-}$, is obtained from the difference between X-ray absorption spectra (XAS) at different helicities of circularly polarized light, μ_{+} and μ_{-}. **c**, XAS/XMCD spectra of H-phase $MoSe_2$ in the $MoSe_2$-$CrSe_2$ lateral heterostructures measured at 78 K.

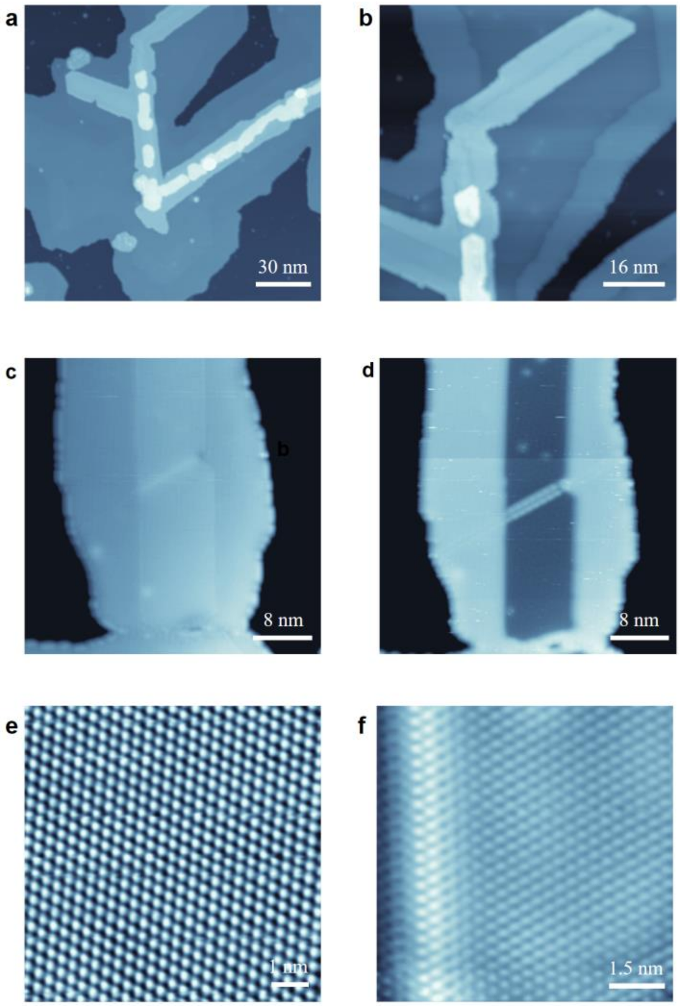

Supplementary Figure 5 | STM characterization of $MoSe_2$-$CrSe_2$ lateral heterostructures. **a**, Large-scale STM image of the $MoSe_2$-$CrSe_2$ lateral heterostructures (V_S = 2.2 V, I_t = 10 pA). **b**, Close-up STM image of the $MoSe_2$-$CrSe_2$ heterostructures (V_S = 1.3 V, I_t = 30 pA). Bias-voltage dependent STM image contrast between the $CrSe_2$ and $MoSe_2$ segments in **c** (V_S = 1.5 V, I_t = 10 pA) and **d** (V_S = −1.5 V, I_t = 10 pA). **e**, Atomic-resolution STM image of the single-layer $CrSe_2$ in the heterostructures (V_S = 0.03 V, I_t = 600 pA). **f**, Atomic-resolution STM image of the second-layer $CrSe_2$ on the $MoSe_2$ interlayer (V_S = −0.05 V, I_t = 1.2 nA). There is no moiré Morie pattern observed in the second-layer $CrSe_2$, indicating the atomically matched lattice with the underlying $MoSe_2$ layer.

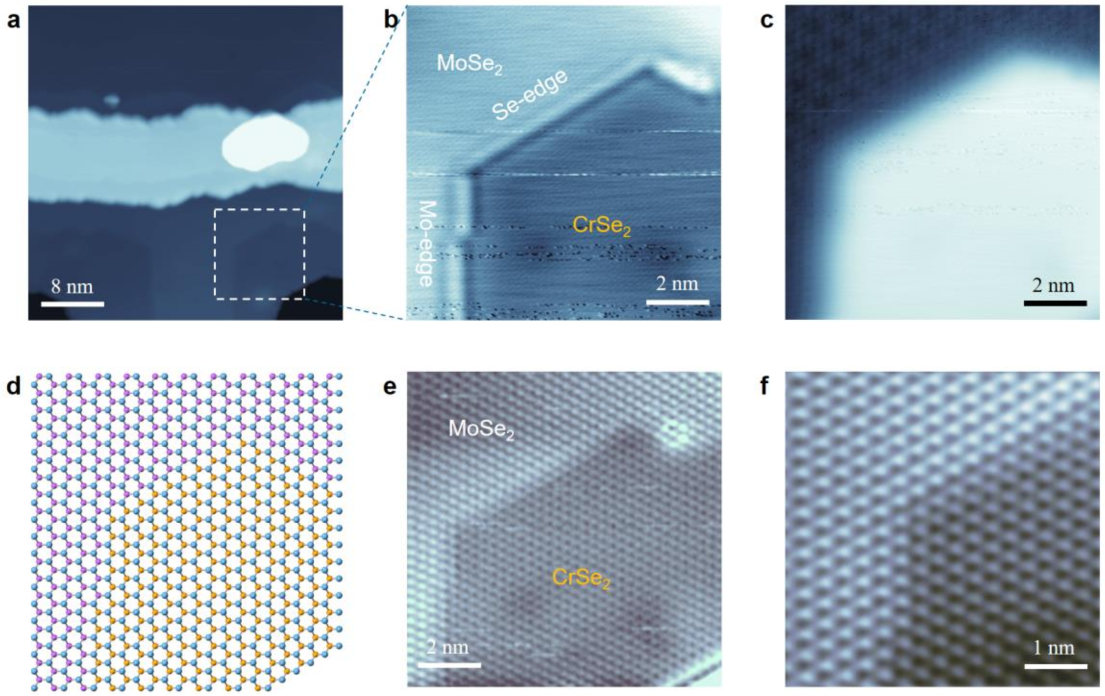

Supplementary Figure 6 | Interfacial structures of $MoSe_2$-$CrSe_2$ lateral heterostructures. **a**, STM image of the $MoSe_2$-$CrSe_2$ lateral heterostructures exhibiting the adjacent Mo-edge and Se-edge with the angle of 120° (V_S = 1.8 V, I_t = 5 pA). **b**, Close-up STM image of the heterostructure interface (V_S = 1.4 V, I_t = 10 pA). **c**, The different STM image contrast between $CrSe_2$ and $MoSe_2$ at the same area (V_S = −1.0 V, I_t = 10 pA). **d**, The ball-and-stick model diagram of the heterostructure interfaces. **e**, Constant-height nc-AFM image of the corresponding $MoSe_2$-$CrSe_2$ interfaces (Tip height z = −360 pm relative to the height at the setpoint 1.3 V, 10 pA). **f**, Close-up nc-AFM image exhibiting the lattice-matched interfacial structures at the Mo-edge and Se-edge (Tip height z = −360 pm relative to the height at the setpoint 1.3 V, 10 pA).

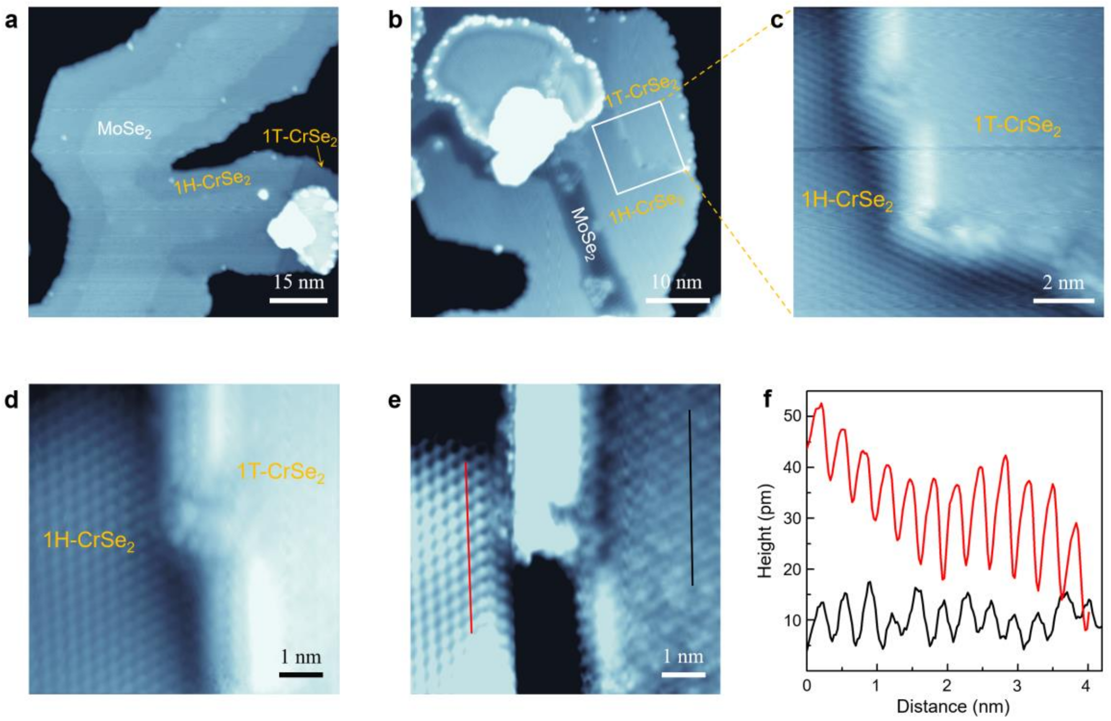

Supplementary Figure 7 | STM images of 1H-1T CrSe2 boundaries. a, STM image of the $MoSe_2$-$CrSe_2$ lateral heterostructure with 1H-1T $CrSe_2$ boundaries (V_S = 2 V, I_t = 10 pA). The 1H and 1T phases can be distinguished by the different surface morphologies due to the different electronic properties. **b**, STM image of the heterostructure with 1H-1T $CrSe_2$ boundaries (V_S = −1.2 V, I_t = 80 pA). **c**, Close-up STM image of the 1H-1T $CrSe_2$ interface (V_S = −0.6 V, I_t = 300 pA). As a result of the different phase structures and lattice constants between 1H and 1T phases, the rough interfaces with misfit dislocations can be observed. **d**, Atomic-resolution STM image of the 1H-1T $CrSe_2$ interface (V_S = −0.3 V, I_t = 600 pA). **e**, STM image of the same area in **d** at the different bias voltage (V_S = −0.1 V, I_t = 600 pA). **f**, Height profile taken along the red and black lines in **e**.

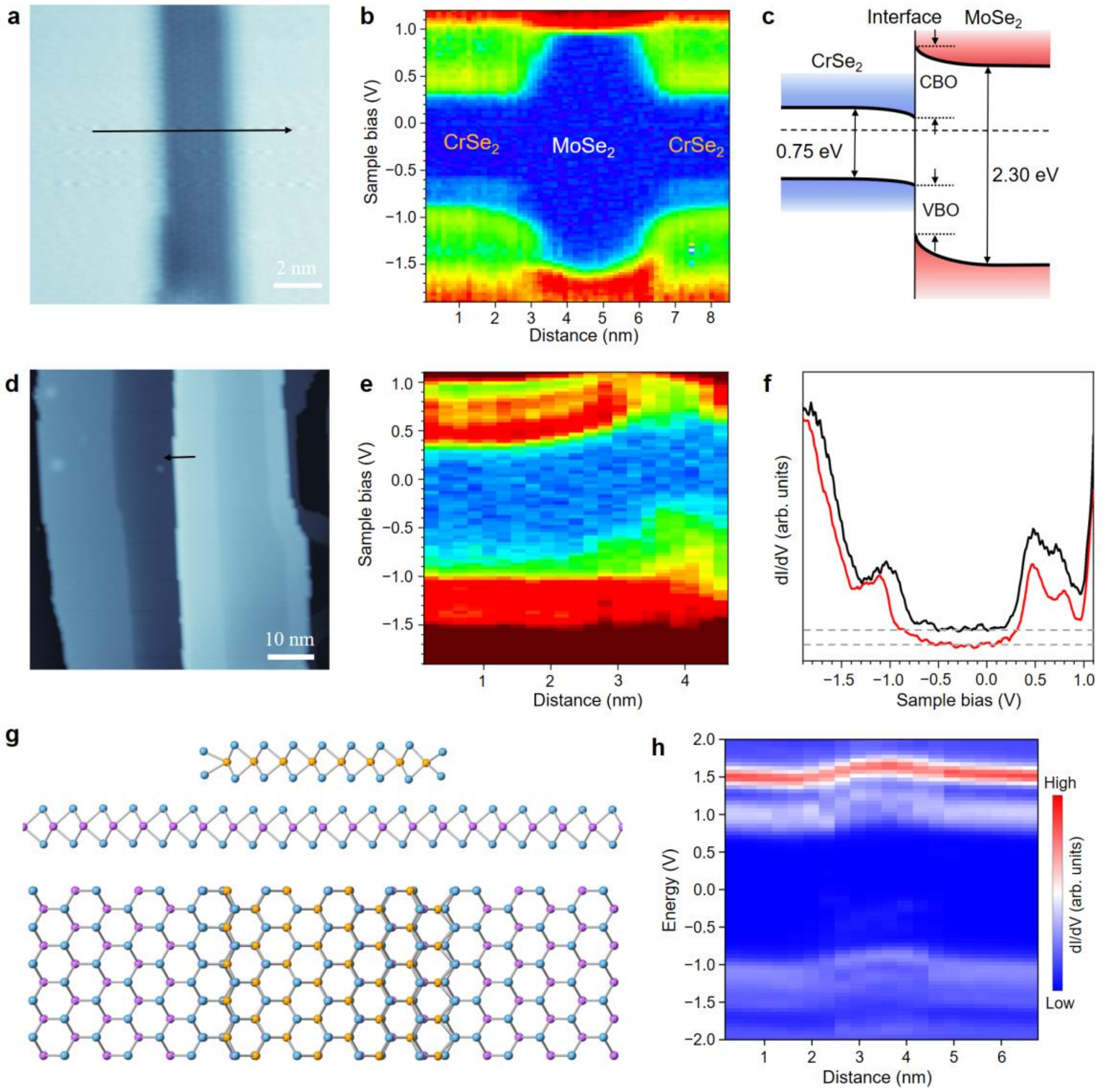

Supplementary Figure 8 | Band bending at the interfaces of $MoSe_2$-$CrSe_2$ lateral and $CrSe_2$/$MoSe_2$ vertical heterostructures. a, STM image of the $MoSe_2$-$CrSe_2$ lateral heterostructures with about 4 nm-width $MoSe_2$ nanoribbon in between (V_S = 1.5 V, I_t = 10 pA). **b**, 2D plot of the dI/dV spectra across the heterostructure interfaces along the along the black arrow in **a**. **c**, Schematic diagram of band alignments in the $CrSe_2$-$MoSe_2$ lateral heterostructure labelled with the CBO and VBO. **d**,The different STM image contrast between $CrSe_2$ and $MoSe_2$ at the same area (V_S = − 1.0 V, I_t = 10 pA). **d**, STM image of the $CrSe_2$/$MoSe_2$vertical heterostructure formed with the second-layer $CrSe_2$ and first-layer $MoSe_2$ in the bilayer structure (V_S = −1.3 V, I_t = 30 pA). **e**, The magnified band profile in the second-layer $CrSe_2$ region. **f**, dI/dV spectra of the second-layer $CrSe_2$ on the $MoSe_2$ interlayer (red) and the first-layer $CrSe_2$ on the HOPG substrate (black). **g**, Atomic model adopted in the DFT calculation with the finite-width $CrSe_2$ nanorribon on $MoSe_2$ substrate. **h**, DFT-calculated LDOS plot of the $MoSe_2$ surface in the $CrSe_2$/$MoSe_2$ vertical heterostructure in which the upward band bending is not localized at vicinity of edges but exists at the whole $MoSe_2$ area under the $CrSe_2$ nanoribbon. The band bending in the first-layer MoSe2 is mainly due to the interlayer charge transfer.

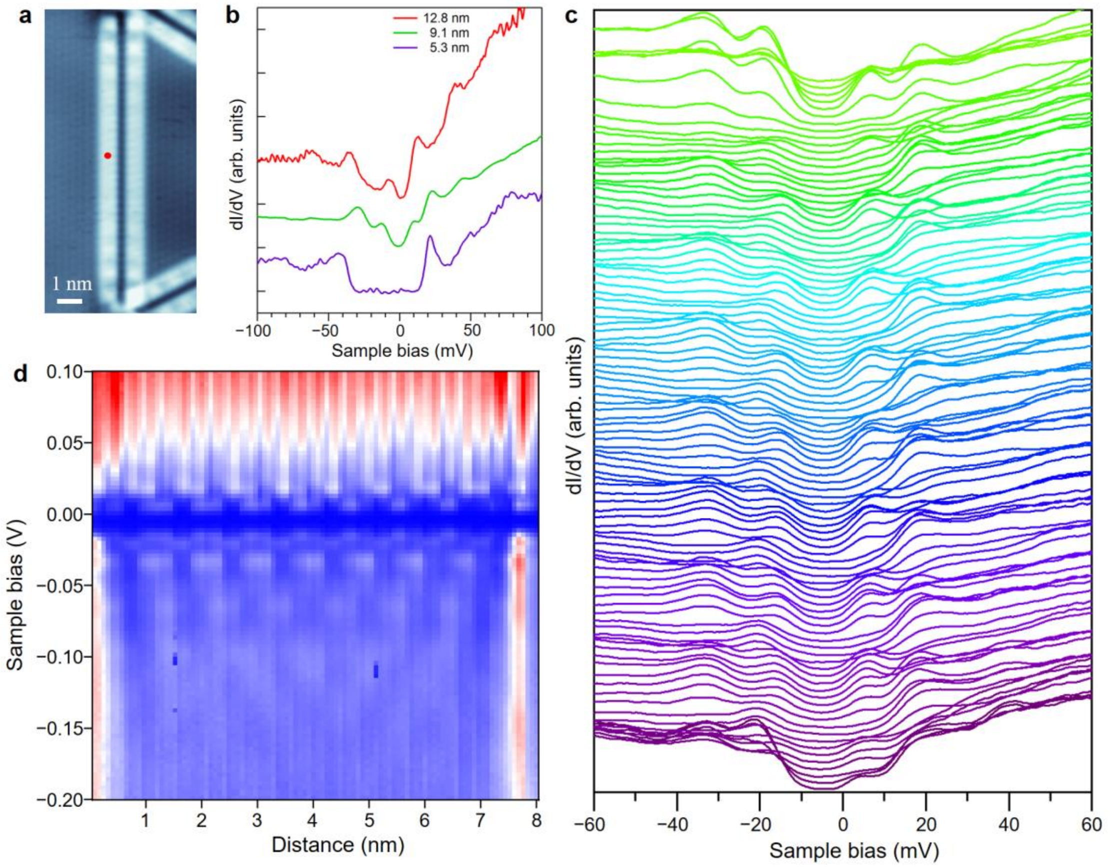

Supplementary Figure 9 | Electronic properties of MTBs in the H-phase $CrSe_2$ monolayer. a, High-resolution STM image of the MTB with a length of ~12.8 nm (V_S = 0.2 V, I_t = 10 pA). **b**, The d*I*/d*V* spectra taken on the MTB with different length. **c**, d*I*/d*V* spectra taken along the MTB with a length of ~9.1 nm. **d**, Real-space imaging of band profile of the MTB with a length of ~9.1 nm.

Supplementary References

1. Chen, Y. et al. Fabrication of $MoSe_2$ nanoribbons via an unusual morphological phase transition. *Nat. Commun.* **8**, 1–9 (2017).

2. Poh, S. M. et al. Large area synthesis of 1D-$MoSe_2$ using molecular beam epitaxy. *Adv. Mater.* **29**, 1605641 (2017).

3. Cheng, F. et al. Controlled growth of 1D $MoSe_2$ nanoribbons with spatially modulated edge states. *Nano Lett.* **17**, 1116–1120 (2017).

4. Xia, Y. et al. Charge density modulation and the Luttinger liquid state in $MoSe_2$ mirror twin boundaries. *ACS Nano* **14**, 10716–10722 (2020).

5. Zhang, C., Johnson, A., Hsu, C.-L., Li, L.-J. & Shih, C.-K. Direct imaging of band profile in single layer MoS_2 on graphite: quasiparticle energy gap, metallic edge states, and edge band bending. *Nano Lett.* **14**, 2443–2447 (2014).

6. Batzill, M. Mirror twin grain boundaries in molybdenum dichalcogenides. *J. Phys. Condens. Matter* **30**, 493001 (2018).

7. Jolie, W. et al. Tomonaga-Luttinger liquid in a box: electrons confined within MoS_2 mirror-twin boundaries. *Phys. Rev. X* **9**, 011055 (2019).
